# Supplementary material for: The Shu complex interacts with the replicative helicase to prevent mutations and aberrant recombination
Source: EMBO J. 2025 Jan 21;44(5):1512–39. doi: 10.1038/s44318-025-00365-9 (PMC11876325; doi:10.1038/s44318-025-00365-9)
Supplement: Supplementary file 1 — Appendix [file 44318_2025_365_MOESM1_ESM.pdf]

Appendix for

The Shu complex interacts with the replicative helicase to prevent mutations and aberrant recombination

Adeola A. Fagunloye[1,*], Alessio De Magis[2,3*], Jordan H. Little[4], Isabela Contreras[1], Tanis J. Dorwart[1], Braulio Bonilla[5], Kushol Gupta[1], Nathan Clark[4,6], Theresa Zacheja[3], Katrin Paeschke[2,3*], Kara A. Bernstein[1,*]

[1]University of Pennsylvania, School of Medicine, Department of Biochemistry and Biophysics, Philadelphia, PA 19104.

[2]Department of Oncology, Hematology and Rheumatology, University Hospital Bonn, Bonn, Germany.

[3]Department of Clinical Chemistry and Clinical Pharmacology, University Hospital Bonn, Bonn, Germany.

[4]University of Utah, Department of Human Genetics, Salt Lake City, Utah 84112.

[5]University of Pittsburgh, School of Medicine, Department of Pharmacology and Chemical Biology, Pittsburgh, PA 15213.

[6]University of Pittsburgh, Department of Biological Sciences, Pittsburgh, PA 15260.

*Equal contribution

Correspondence: kpaeschk@uni-bonn.de (K.P); kara.bernstein@pennmedicine.upenn.edu (K.B)

TABLE OF CONTENTS

Appendix Figure S1: Psy3 N-terminal mutants are expressed, and total recombination was assessed using a direct repeat recombination assay..2

Appendix Figure S1

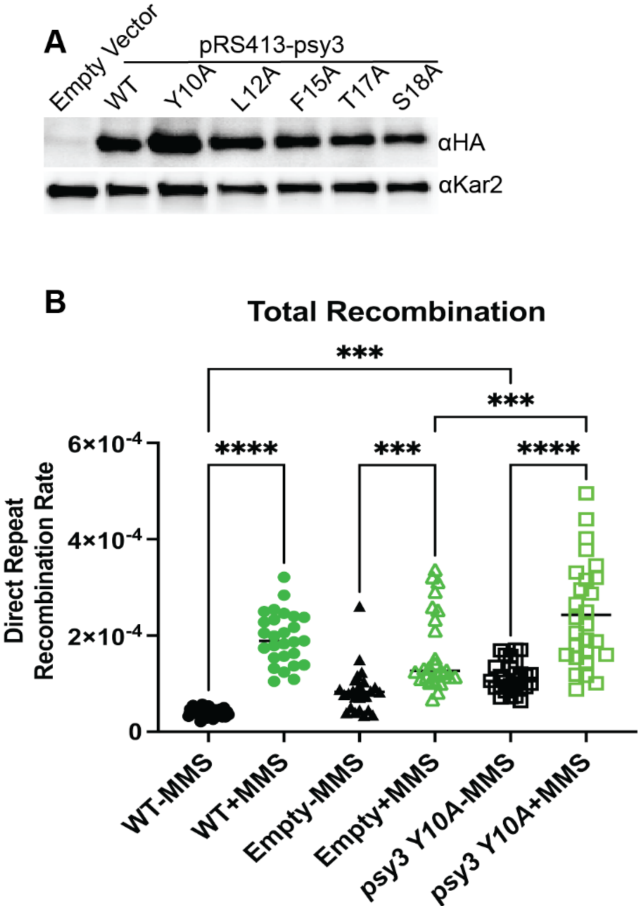

Appendix Figure S1: Psy3 N-terminal mutants are expressed, and total recombination was assessed using a direct repeat recombination assay.

(**A**) Protein expression of HA-tagged WT Psy3, psy3 N-terminal mutants (Y10A, L12A, F15A, T17A, and S18A) expressed in a pRS413 vector was accessed by western blot. Protein was isolated by TCA precipitation from untagged WT or *psy3* mutants' cells expressing the HA-tag. The Psy3 protein levels were assessed by western blot (αHA) or for equal protein loading (αKar2). (**B**) WT, *psy3*Δ or *psy3-Y10A* expressing cells harboring a direct repeat HR reporter (*leu2-ΔEcoRI::URA3::leu2-ΔBstEII*) were tested for spontaneous total recombination rates and upon 0.0003% MMS exposure as described (0.0003%, 18 hours). Exact p values (WT-MMS vs. WT+MMS p=≤ 0.0001, empty - MMS vs. empty + MMS p=0.0002, *psy3-Y10A*-MMS vs. *psy3-Y10A* +MMS p=≤ 0.0001, empty + MMS vs. *psy3-Y10A*+MMS p=0.0001, WT-MMS vs. *psy3-Y10A*-

MMS $p=0.0005$). Nine independent colonies were measured for each experiment, and the median value from three experiments (horizontal bar) were plotted. The Tukey test determined significance where *** represents $p \leq 0.001$ and **** represents $p \leq 0.0001$.
