# Supplementary figures and images for: The Shu complex interacts with the replicative helicase to prevent mutations and aberrant recombination
Source: EMBO J. 2025 Jan 21;44(5):1512–39. doi: 10.1038/s44318-025-00365-9 (PMC11876325; doi:10.1038/s44318-025-00365-9)

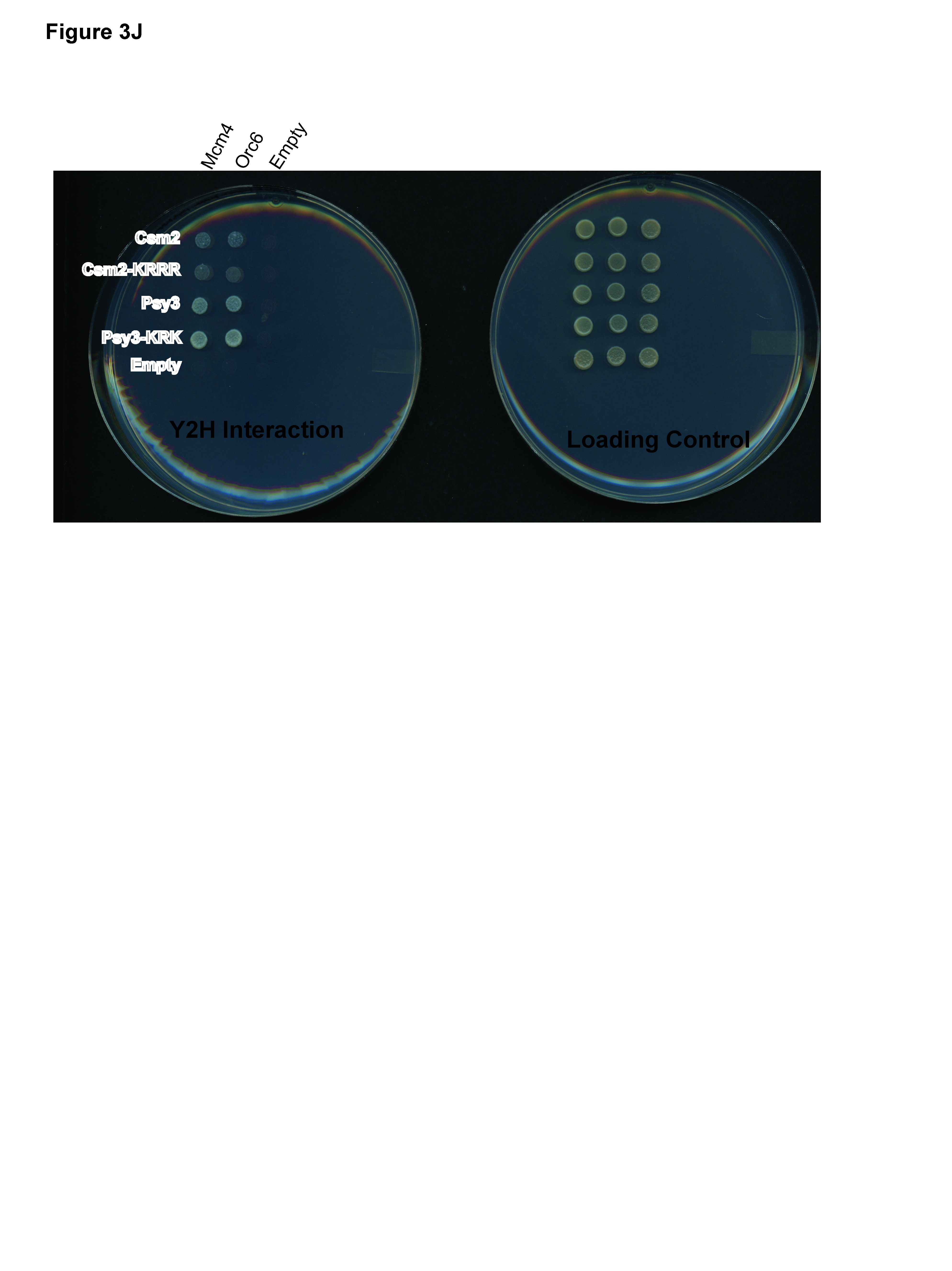

Supplement: Supplementary file 5 — Source data Fig. 3 [file 44318_2025_365_MOESM5_ESM.zip › Figure 3/3J/Fig3 J.tiff]

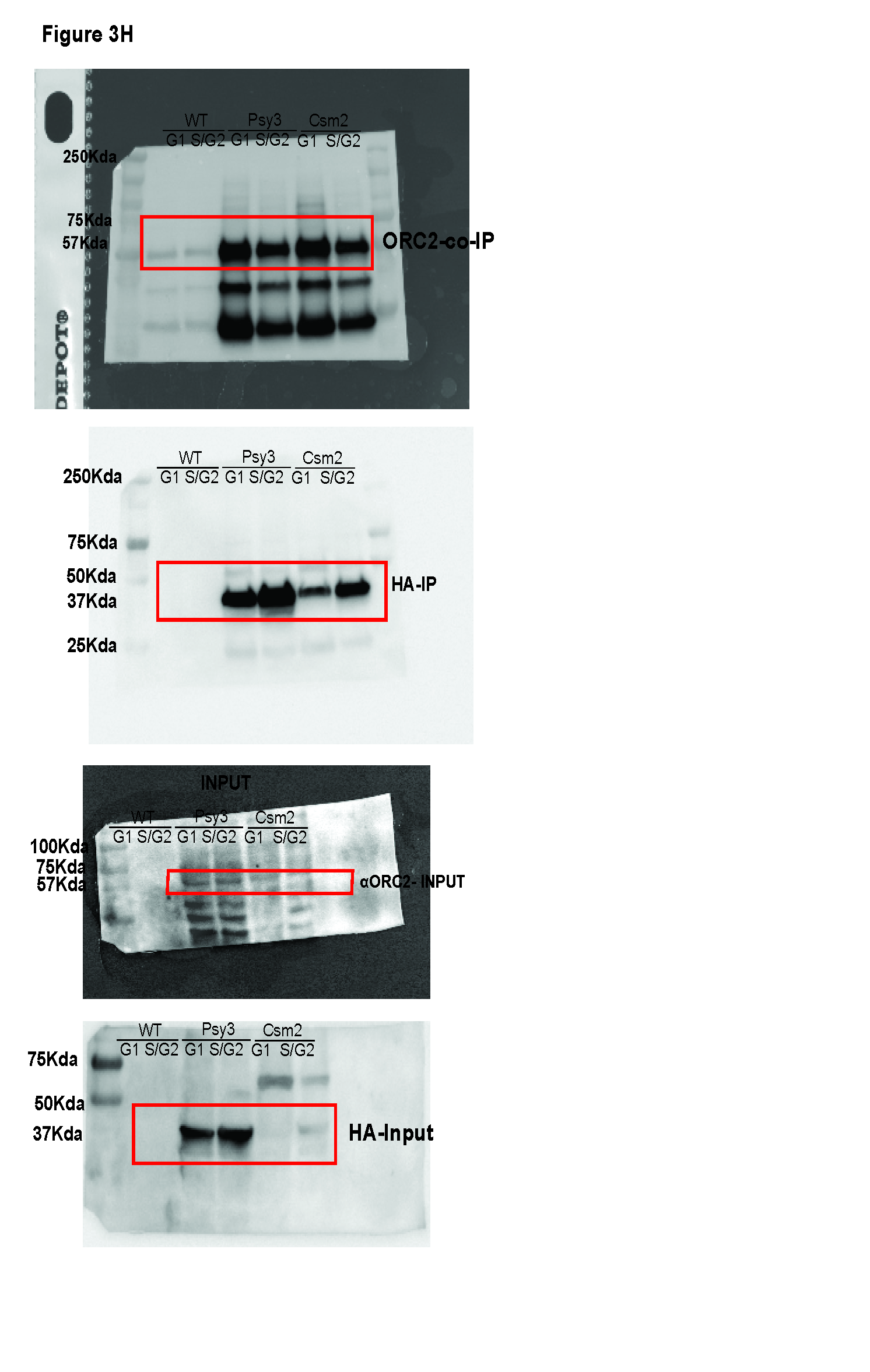

Supplement: Supplementary file 5 — Source data Fig. 3 [file 44318_2025_365_MOESM5_ESM.zip › Figure 3/3H/Figure 3H.tiff]

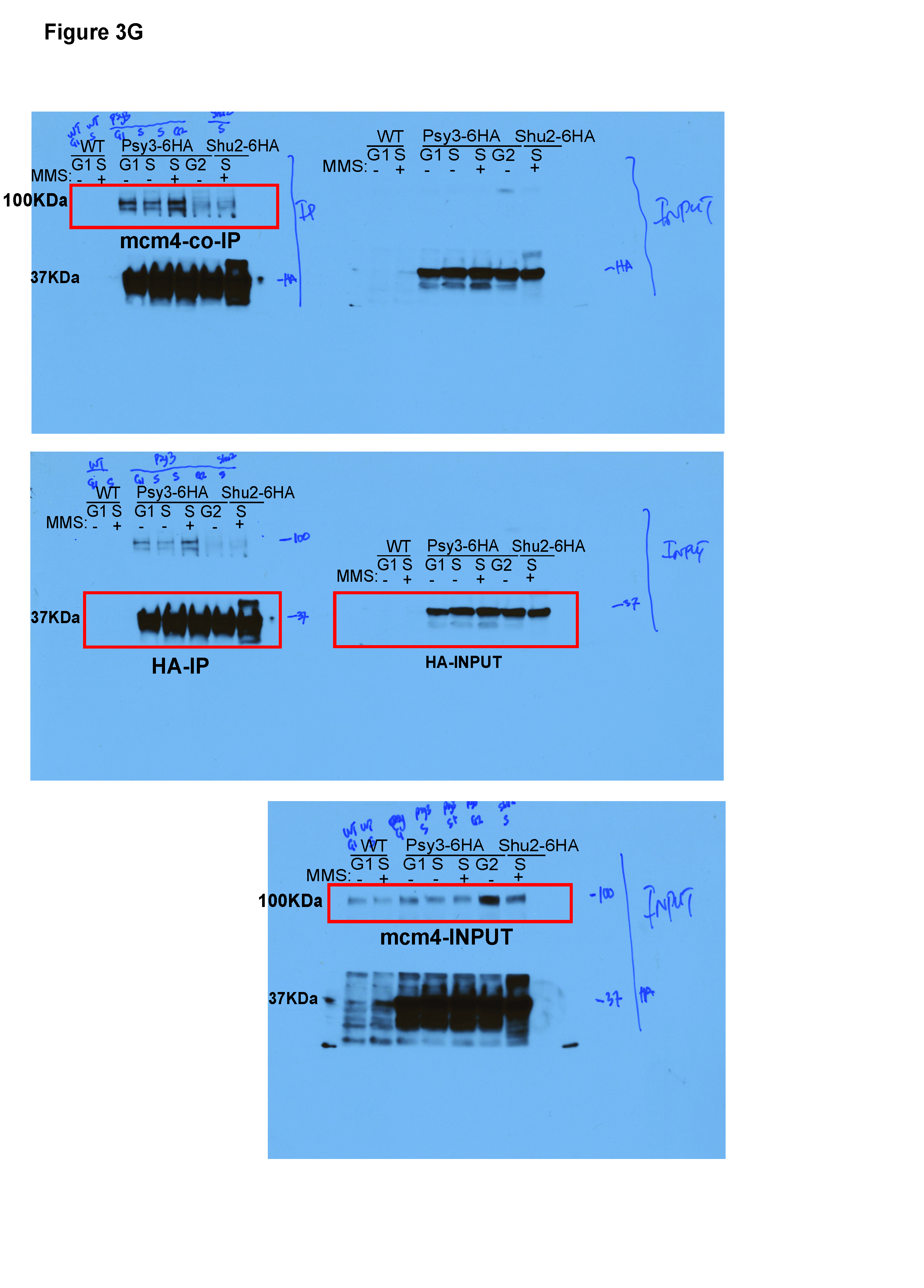

Supplement: Supplementary file 5 — Source data Fig. 3 [file 44318_2025_365_MOESM5_ESM.zip › Figure 3/3G/Fig 3G.tiff]

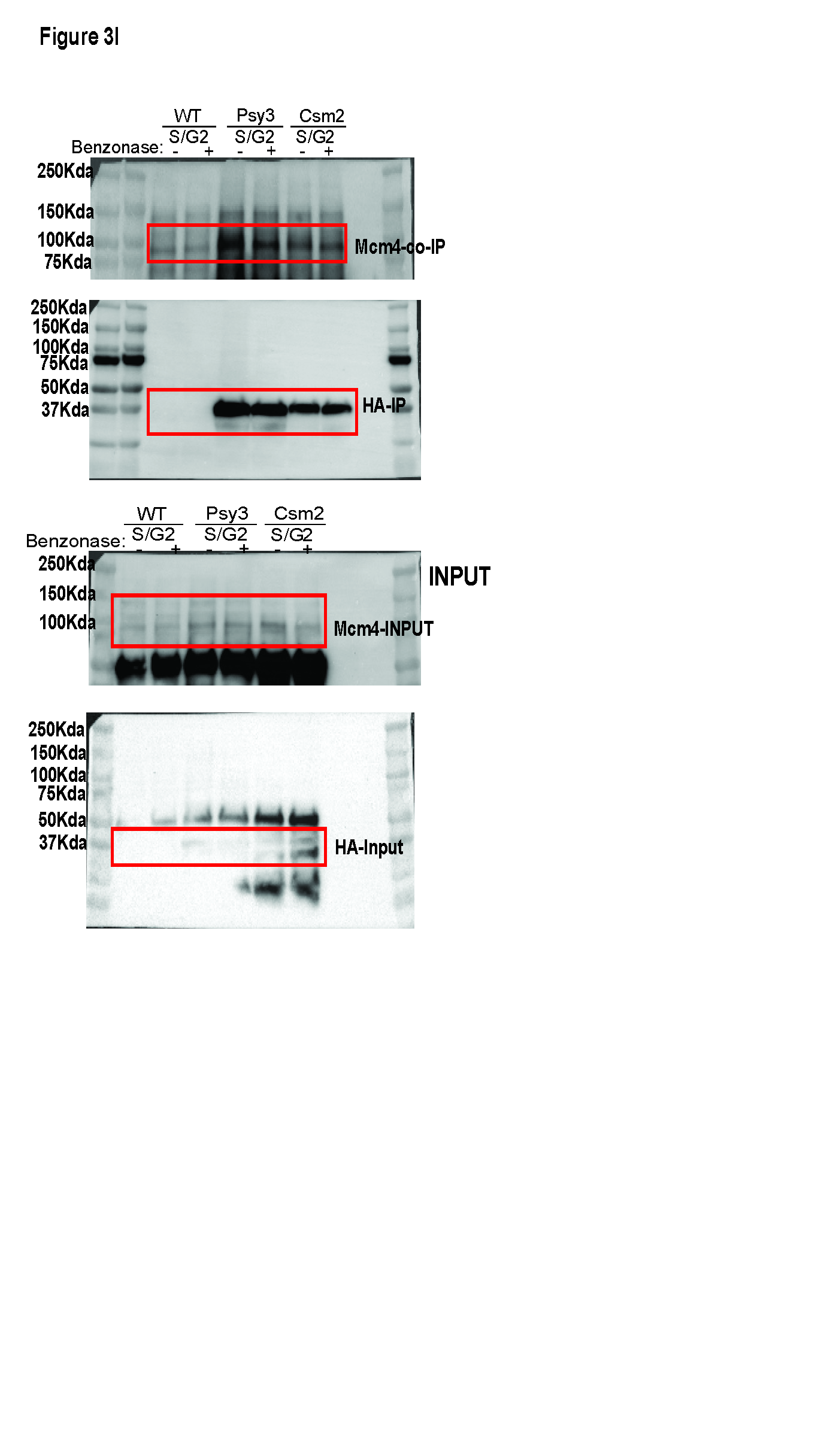

Supplement: Supplementary file 5 — Source data Fig. 3 [file 44318_2025_365_MOESM5_ESM.zip › Figure 3/3I/Figure3I.tiff]

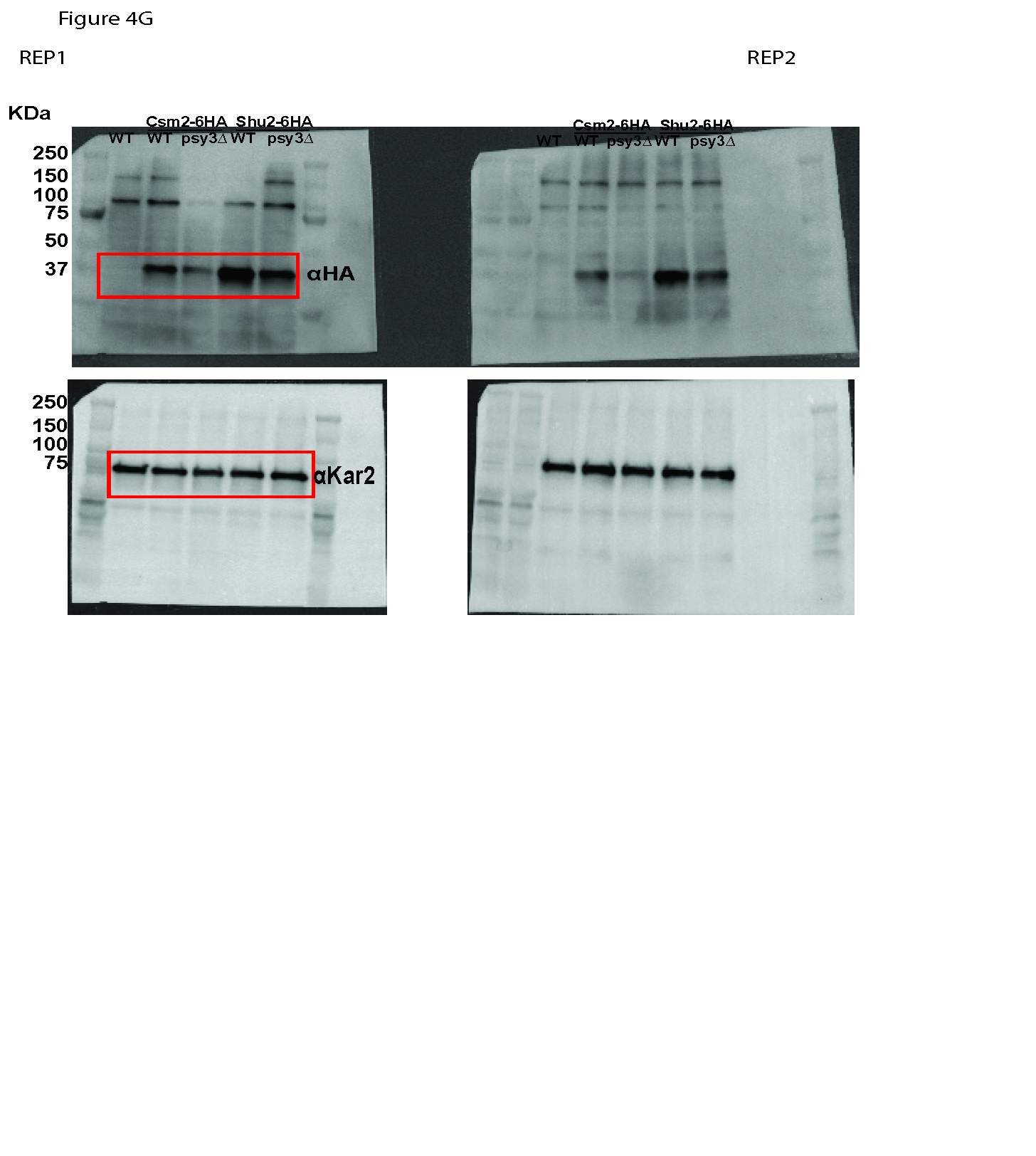

Supplement: Supplementary file 6 — Source data Fig. 4 [file 44318_2025_365_MOESM6_ESM.zip › Figure 4/Fig 4G/Fig 4G.tiff]

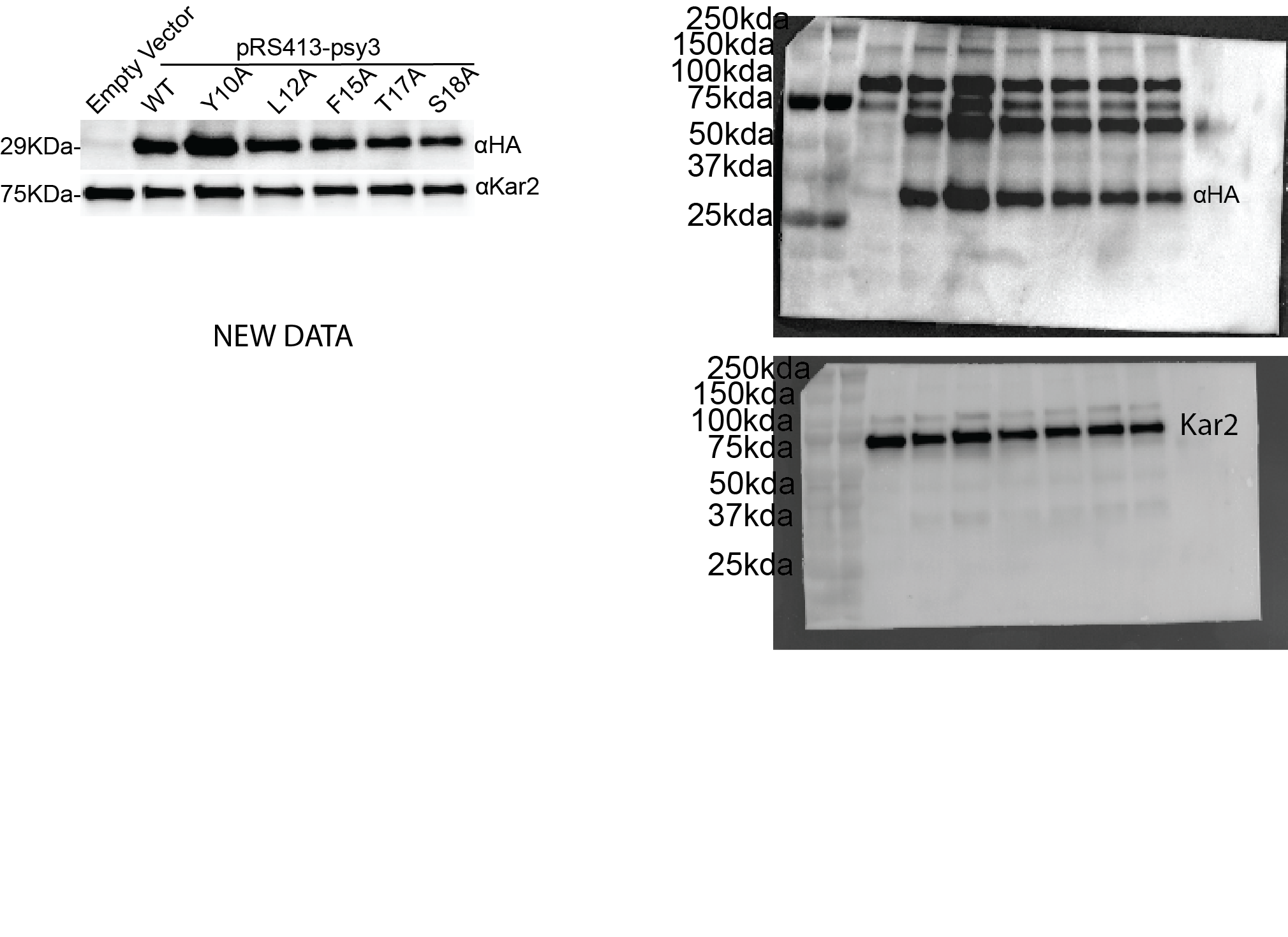

Supplement: Supplementary file 9 — EV and Appendix Figure Source Data [file 44318_2025_365_MOESM9_ESM.zip › EV and S1 Fig/Fig S1/Fig S1 A.png]
