# Supplementary material for: The Shu complex interacts with the replicative helicase to prevent mutations and aberrant recombination
Source: EMBO J. 2025 Jan 21;44(5):1512–39. doi: 10.1038/s44318-025-00365-9 (PMC11876325; doi:10.1038/s44318-025-00365-9)
Supplement: Supplementary file 10 — Expanded View Figures [file 44318_2025_365_MOESM10_ESM.pdf]

Expanded View Figures

Figure EV1. Csm2, Rad55, and Rad52 significantly overlap at the same DNA binding regions by ChIP seq.

(**A**) Five-fold serial dilution of the parental wild-type strain and strains expressing either Csm2-6HA or Rad55-9MYC on rich YPD medium or YPD medium containing the indicated concentration of MMS. (**B**) Genome-wide correlation of the Csm2 peaks with the RAD55 peaks. Exact *p*-value: $p = < 0.0001$. (**C**) Genome-wide correlation of the RAD55 peaks with the ARS sites. Exact *p*-value: $p = 0.36$. (**D**) Genome-wide correlation of the Csm2 peaks with the RAD52 peaks (Costantino and Koshland, 2018). Exact *p*-value: $p = < 0.0001$. (**E**) Genome-wide correlation of the RAD55 peaks with the RAD52 peaks (Costantino and Koshland, 2018). Exact *p*-value: $p = < 0.0001$. (**B–E**) Significance is assessed by genome-wide permutation test (**F**) IGV Genome Browser screenshot of genome-wide Csm2, RAD55, RAD52, (Costantino and Koshland, 2018), MCM2-7 binding sites at ChrII:611,265-613,215 (Lee et al, 2021). ARS database is from OriDB, produced in Saccer1. Source data are available online for this figure.

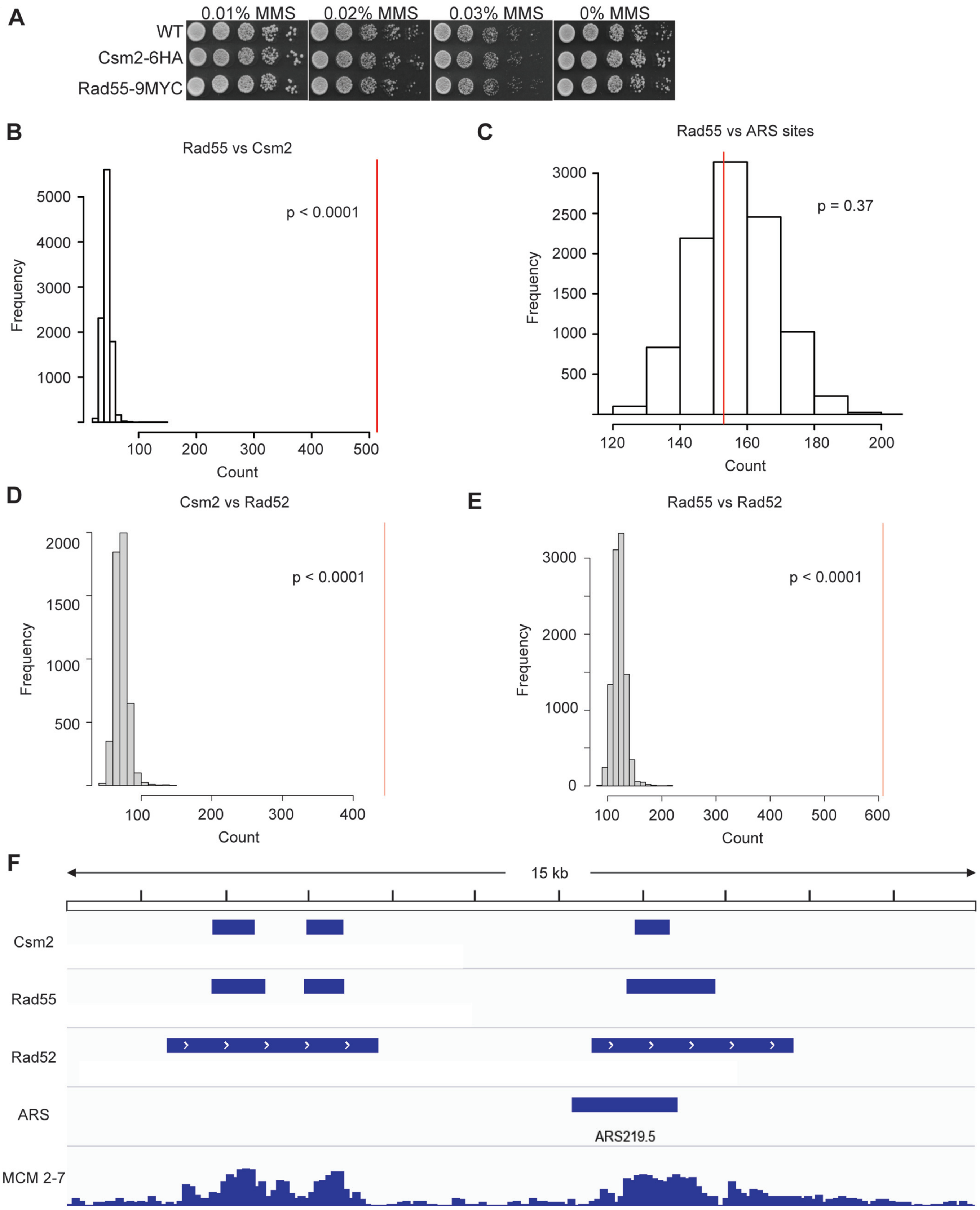
A
0.01% MMS
0.02% MMS
0.03% MMS
0% MMS
WT
Csm2-6HA
Rad55-9MYC
B
Rad55 vs Csm2
p < 0.0001
Frequency
Count
C
Rad55 vs ARS sites
p = 0.37
Frequency
Count
D
Csm2 vs Rad52
p < 0.0001
Frequency
Count
E
Rad55 vs Rad52
p < 0.0001
Frequency
Count
F
15 kb
Csm2
Rad55
Rad52
ARS
ARS219.5
MCM 2-7

 © The Author(s)

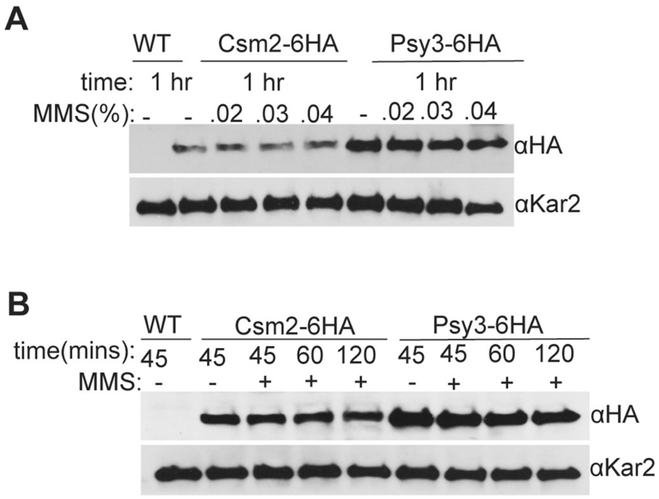

Figure EV2. Csm2 and Psy3 steady-state protein levels are maintained upon MMS exposure.

(**A**) Csm2 and Psy3 steady-state protein levels are not increased upon MMS exposure. Csm2-6HA or Psy3-6HA expressing strains were exposed to the indicated dose of MMS for one hour and then protein levels were accessed by western blot using αHA or αKar2 antibodies. Kar2 was used as a loading control. (**B**) Csm2 and Psy3 steady-state protein levels remain constant over time after 0.03% MMS exposure. Csm2-6HA or Psy3-6HA expressing strains were exposed to 0.03% MMS for the indicated amount of time (45, 60, or 120 min) and then protein levels were accessed by western blot using αHA or αKar2 antibodies. Kar2 was used as a loading control. Source data are available online for this figure.

© The Author(s)

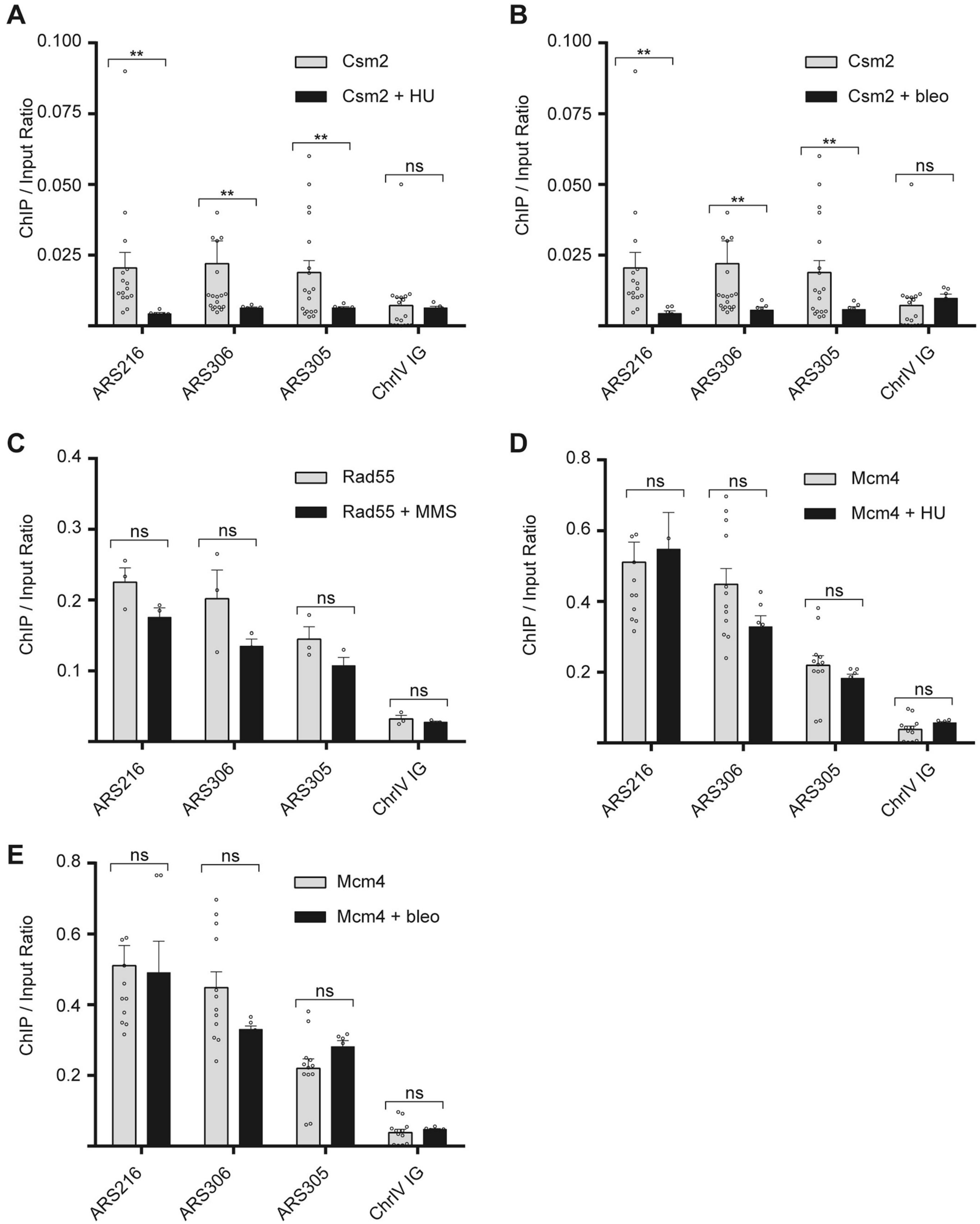
A
B
C
D
E
ChIP / Input Ratio
Csm2
Csm2 + HU
Csm2 + bleo
Rad55
Rad55 + MMS
Mcm4
Mcm4 + HU
Mcm4 + bleo
**
ns
0.100
0.075
0.050
0.025
0.8
0.6
0.4
0.2
0.3
0.1
ARS216
ARS306
ARS305
ChrIV IG

 © The Author(s)

Figure EV3. Unlike Rad55 or Mcm4, Csm2 enrichment at ARS sites is reduced upon DNA damage.

(**A**) ChIP and qPCR of Csm2-6HA untreated or treated for 2 h with 100 mM HU. Exact *p*-value: ARS216 $p = 0.007797$, ARS305 $p = 0.001494$, ARS306 $p = 0.006935$, and ChrIV IG $p = 0.464314$. (**B**) ChIP and qPCR of Csm2-6HA untreated or treated for 2 h with 20 ng/µL bleomycin. Exact *p*-value: ARS216 $p = 0.002339$, ARS305 $p = 0.001465$, ARS306 $p = 0.003030$, ChrIV IG $p = 0.164196$. (**C**) ChIP and qPCR of RAD55-9MYC untreated or treated for 2 h with 0.02% MMS. Exact *p*-value: ARS216 $p = 0.163698$, ARS305 $p = 0.106164$, ARS306 $p = 0.051302$, and ChrIV IG $p = 0.307960$. (**D**) ChIP and qPCR of MCM4 untreated or treated for 2 h with 100 mM HU. Exact *p*-value: ARS216 $p = 0.386663$, ARS305 $p = 0.295602$, ARS306 $p = 0.089571$ and ChrIV IG $p = 0.327584$. (**E**) ChIP and qPCR of MCM4 untreated or treated for 2 h with 20 ng/µL bleomycin. Exact *p*-value: ARS216 $p = 0.280850$, ARS305 $p = 0.087874$, ARS306 $p = 0.188565$ and ChrIV IG $p = 0.446871$. For all experiments, qPCRs were performed at three ARS sites (ARS302, ARS306, ARS305) and one control (ChrIV IG). All plotted results were based on the ChIP/Input ratio average of at least three independent experiments ± SEM. Significance was calculated based on a one-sided Student's t-test. Asterisks indicate statistical significance in comparison with wild-type cells under the same experimental conditions. *$p < 0.05$, **$p < 0.01$ and ns = not statistically significant. Source data are available online for this figure.

© The Author(s)

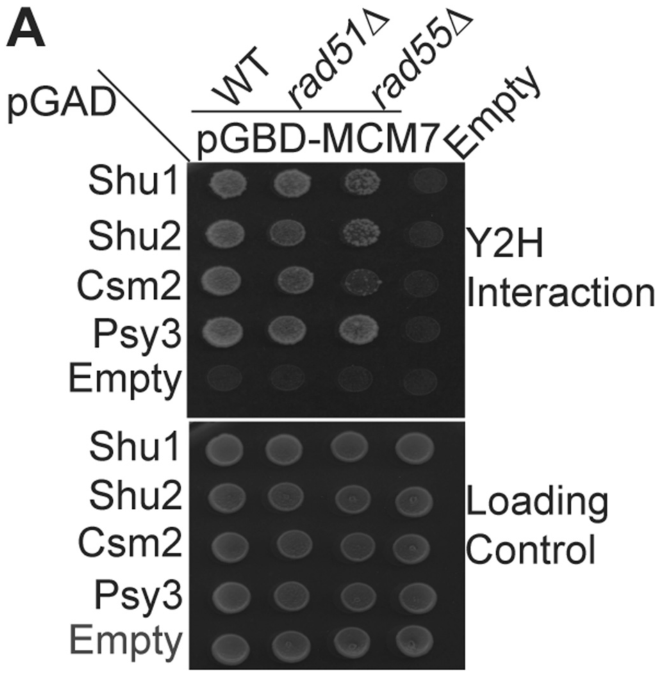

Figure EV4. Mcm7 Y2H interaction with the Shu complex is independent of *RAD51* or *RAD55*.

Shu complex Y2H interaction with MCM complex member (Mcm7) is independent of *RAD51* and *RAD55*. Y2H experiment examining Shu complex (Shu1, Shu2, Csm2, Psy3) interaction with Mcm7 transformed in Y2H strain with either *rad51*Δ or *rad55*Δ knocked out. Yeast with the indicated plasmids were grown in SC-L-W, plated on SC-L-W-H medium, and incubated for 2–3 days at 30 °C. Empty vectors are negative controls. Growth is indicative of a Y2H interaction and SC-L-W is used as a loading control. All experiments were done in triplicate. Source data are available online for this figure.

 © The Author(s)

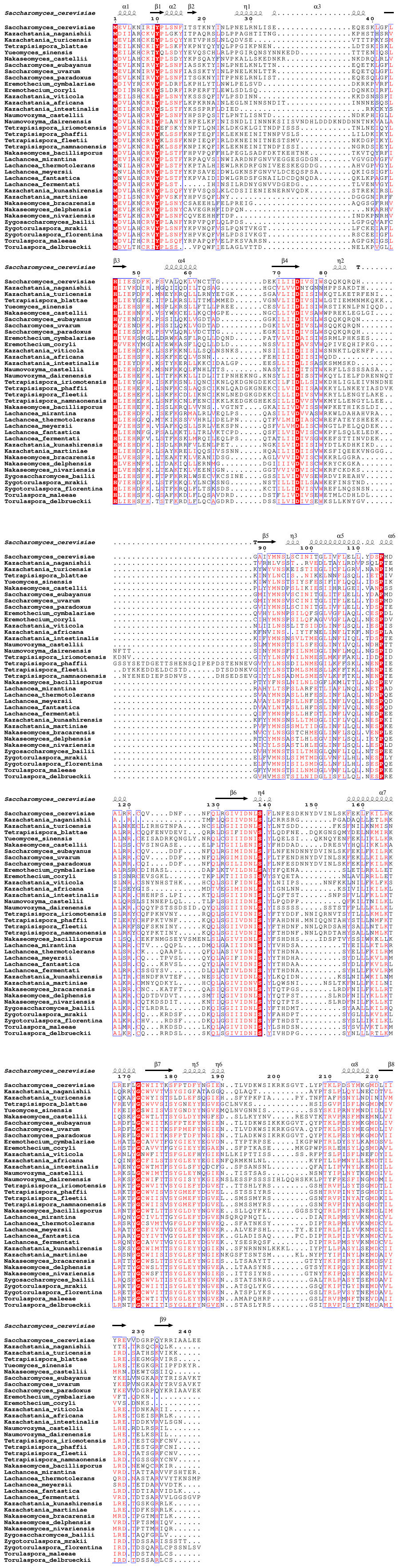

Figure EV5. Psy3 sequence alignment from 36 fungi species.

Sequence alignment of Shu complex member, Psy3, from the indicated 36 fungi species (Larkin et al, 2007). Invariant residues are shown in red boxes, and similar residues are shown in red text and outlined in blue. The predicted protein folding based on the *S. cerevisiae* structure (PDB: 5XYN) (Zhang et al, 2017) is shown above. The figure was made with ESPript3 (Robert and Gouet, 2014).

 © The Author(s)
